# Applying Constructive Alignment to Enhance the Educational Structure of the European Society for Vascular Surgery Podcasts

**DOI:** 10.1016/j.ejvsvf.2026.02.002

**Published:** 2026-02-13

**Authors:** Carlota F. Prendes, Laurence Bertrand, Vaiva Dabravolskaite, Suzanne Stokmans, Egle Kavaliunaite, Melina Vega de Ceniga

**Affiliations:** a Department of Surgical Sciences, Uppsala University Hospital, Uppsala, Sweden; b Vascular Surgery Department, Ludwig Maximilian University Hospital, Munich, Germany; c Inselspital, University Hospital Bern, Bern, Switzerland; d Isala Hospital, Zwolle, the Netherlands; e Department of Cardiac, Thoracic and Vascular Surgery, Odense University Hospital (OUH), Odense, Denmark; f Department of Angiology and Vascular Surgery, University Hospital of Galdakao-Usansolo, University of the Basque Country (EHU), Bizkaia, Spain

**Keywords:** ESVS, Medical education, Podcasts, Social media, Vascular surgery

## Abstract

**Objective:**

Medical podcasts are increasingly being recognised as flexible, accessible tools for lifelong learning. However, their educational structure and cognitive demands often remain informal and unstandardised. This study aimed to evaluate the educational quality of the European Society for Vascular Surgery (ESVS) vascular surgery podcasts using pedagogical frameworks and propose enhancements to improve their impact.

**Methods:**

A mixed methods educational design was employed, structured around three core components: an exploratory online survey guided by the Kirkpatrick Evaluation Model; application of Bloom's Revised Taxonomy to analyse three selected podcast episodes; and creation of pedagogical enhancements for them according to constructive alignment principles. The primary outcome was to identify areas where targeted improvements could enhance their overall educational impact.

**Results:**

Fifty-seven participants completed the online survey. Over 70% indicated that the podcasts had improved their understanding of vascular surgery topics (Kirkpatrick Level 2) and 75% reported that they had applied podcast content in practice (Kirkpatrick Level 3). Content analysis of the selected podcast episodes revealed good alignment with Bloom's lower order cognitive skills (remembering, understanding, applying), with occasional support for higher order domains (analysing, evaluating, creating). Potential educational enhancements also supported by survey responders included brief episode summaries (68%), links to further readings and references (51%), and inclusion of intended learning objectives (37%). These enhancements were categorised as low to moderate effort with high potential educational impact.

**Conclusion:**

The ESVS podcast series is a valued educational tool. Integration of pedagogically informed features such as intended learning outcomes, summaries, and linked references, could enhance learning outcomes and retention without significantly increasing production demands.

## Highlights

• ESVS podcasts: Bloom/Kirkpatrick show strong alignment with lower order levels.
• Survey (n=57): 73.7% reported clinical application of podcast derived knowledge.
• Potential low-effort, high-impact enhancements: ILOs, summaries, references.

## INTRODUCTION

Murder mysteries, space explorations, financial trends, and top political headlines are among the many subjects explored on some of America's most popular podcasts.^1^^,^^2^ Since *The Guardian* first coined the term “podcast” in 2004, the medium has grown rapidly, offering listeners on the go entertainment.^1^^,^^3^^,^^4^ Beyond leisure, podcasts have also become a powerful tool for lifelong learning, delivering expert insights, up to date information, and deep dives into specialised topics in an accessible and engaging format. Podcasts are also emerging as a valuable tool in medical education, with some medical podcasts being accessed in over 190 countries by medical students, residents, and practicing clinicians.^5^^,^^6^ Their widespread use and perceived educational benefit have led to increasing integration within formal medical training. In the United States, several medical schools and residency programs have incorporated podcasts into structured curricula. For example, the Accreditation Council for Graduate Medical Education (ACGME) allows emergency medicine residents to apply podcast based learning, when accompanied by assessment questions, towards accredited educational hours, with one hour of credit granted per five hours of listening.^5^

The European Society for Vascular Surgery (ESVS) vascular surgery podcasts were established as an educational initiative by the ESVS and the *European Journal of Vascular and Endovascular Surgery: Vascular Forum*.^7^ They began as one of the activities of the revamped ESVS second journal, *EJVES Vascular Forum*. The objective was to expand to a different format of communication and to a larger and younger audience, including audio interviews, recorded critical appraisal pieces, and editorials. Since the beginning of the project in late 2019, the ESVS podcasts have released over 125 episodes, accumulated over 67 000 downloads by > 3 000 different listeners, and have more than 1 080 followers.^8^ Podcast formats include interviews, question and answer (Q&A) series on ESVS guidelines, recorded edutorials published in the EJVES, critical appraisal of EJVES Editor's Choice papers, among others, with various topics including aortic, peripheral, carotid, and venous pathology. All podcasts and formats have been downloaded between 250 and 700 times; they all seem to be accepted, popular, and impactful.

Despite their popularity, their educational attributes have not been formally evaluated. Therefore, this study, carried out by former and current members of the ESVS podcast committee, aimed to evaluate the current educational impact of the ESVS vascular surgery podcasts and suggest potential educational enhancements to serve as a framework for future episodes.

## METHODS

The project employed a mixed methods educational design structured around three core components: an exploratory online survey based on the Kirkpatrick Evaluation Model; the application of Bloom's Revised Taxonomy to podcast content; and the development of constructive alignment principles to propose pedagogical enhancements, including the development of intended learning outcomes (ILOs) and adjunctive educational support for the selected episodes.

The Kirkpatrick Evaluation Model evaluates educational effectiveness across four hierarchical levels: Level 1 – Reaction (learner engagement and satisfaction), Level 2 – Learning (knowledge or skill acquisition), Level 3 – Behaviour (application in practice), and Level 4 – Results (broader educational or clinical impact), allowing assessment of both immediate and sustained learning outcomes.^9^^,^^10^ Bloom's Revised Taxonomy, on the other hand, categorises educational objectives into six progressive levels of cognitive complexity: Remember, Understand, and Apply (lower level cognitive skills); and Analyse, Evaluate, and Create (higher level cognitive skills).^11^ The selection and adaptation of these models for this project were developed in collaboration with an educational specialist to ensure methodological rigor and relevance to digital medical education, and both models were chosen to obtain a complementary perspective.

### Online survey

An online survey was developed on Google Forms to gather formative feedback from ESVS podcast series listeners.^12^ Questions were created with Kirkpatrick Evaluation Model levels as a base.^9^^,^^10^ Survey items included both closed and open ended questions, addressing listener demographics, learner engagement and satisfaction (Level 1); knowledge or skill acquisition (Level 2); application into practice (Level 3); and broader educational or clinical impact (Level 4). The survey was disseminated via ESVS communication channels and selected social media channels (LinkedIn, Twitter, specific vascular surgical WhatsApp groups), with three reminders being sent out. It was open to everybody and ESVS membership was not required.

### Application of Bloom's Taxonomy to podcast content

Bloom's Revised Taxonomy was used to evaluate the cognitive depth of podcast content, assessing whether episodes facilitate basic knowledge acquisition or promote higher order learning processes.^11^ Given the broad diversity of podcast formats and objectives, some primarily designed to engage or entertain, with others having a stronger educational focus, three ESVS podcast episodes considered to have a predominantly educational intent were selected. Differences in format, duration, and pedagogical style were also considered when making this selection. The selected podcasts were: #ESVS2024 – vascular access pre-op planning and patient selection (clinical scenario);^13^ Editor's Choice – radiation protection and pregnancy (literature based discussion);^14^ Q&A ESVS vascular trauma guidelines: Pt 2 (guideline interpretation).^15^

### Constructive alignment and pedagogical enhancements

Finally, the principles of constructive alignment were applied to the selected episodes as a potential framework to enhance their educational impact in the future.^16^ For each episode, explicit ILOs were developed. Furthermore, examples of active learning elements such as clinical prompts, reflective questions, or discussion guides, were also included. Finally, a feasibility mapping approach was applied, taking into consideration the estimated implementation effort (low, moderate, high) and anticipated educational impact.^17^^,^^18^

## RESULTS

### Online survey

Fifty-seven participants completed the survey, of whom the majority were men (61%) and vascular surgeons (90%), and had between zero and five years of professional experience (37%). Most respondents were based in Europe (74%) and Africa (18%). Full demographics can be found in Supplementary Table S1. The overall perceived quality was 3.9out of 5, with 72% of respondents assigning a rating of 4 or 5, while their educational impact was rated at 3.93 out of 5, with 70% of respondents assigning a rating of 4 or 5. An overview of podcast replies according to Kirkpatrick level can be found in Table 1. Supported enhancements for improved retention and sustained learning included brief two minute summaries at the end (70%), availability of links to further reading materials (51%), accompanying ILOs (37%), and visual abstracts (32%) (Table 2).

**Table 1:** Podcast educational impact survey questions and respondents’ (*n* = 57) answers according to Kirkpatrick levels.

Kirkpatrick level	Domain or question	Responses (*n* = 57)
Level 1 – Reaction (learner engagement and satisfaction)	How often do you listen to ESVS podcasts? (1–5 Likert scale, 1: Never, 5: every episode)	5: 30%; 4: 28%; 3: 26%; 2: 11%; 1: 5%
	Do you usually listen to: full episodes or selected segments?	47% full episodes; 51% selected segments only
	Clarity and structure of episodes (1–5 Likert scale, 1: Poor, 5: Excellent)	4: 46%; 5: 21% (Total 4–5: 67%)
	Evidence based information presented effectively? (1–5 Likert scale, 1: Never, 5: Always)	4: 51%; 5: 21% (Total 4–5: 72%)
	Balance between expert opinion and guidelines	Good balance: 95%; Too much opinion: 2%; Not sure or can't evaluate: 4%
	Host engagement (1–5 Likert scale, 1: Not engaging, 5: Very engaging)	4: 51%; 5: 14% (Total 4–5: 65%)
	Guest engagement (1–5 Likert scale, 1: Not engaging, 5: Very engaging)	4: 49%; 5: 18% (Total 4–5: 67%)
	Overall quality of ESVS podcasts (1–5 Likert scale, 1: Poor, 5: Excellent)	5: 33%; 4: 39%; 3: 23%; 2: 5%
Level 2 – Learning (knowledge or skill acquisition)	Improved understanding of vascular topics?	Yes: 74%; Not sure: 21%; No: 5%
	Relevance to clinical practice (Likert scale: 1: Not at all, 5: Extremely)	4–5: 44%; 3: 33%; 2: 12%; 1: 11%
Level 3 – Behaviour (application in practice)	Applied podcast derived knowledge in clinical work?	Yes: 75%; No: 25%
	Podcasts stimulated further learning or reading?	Yes: 79%; No: 21%
Level 4 – Results (broader educational or clinical impact)	Overall educational value and impact (1–5 Likert scale, 1: Poor, 5: Excellent)	5: 30%; 4: 40%; 3: 25%; 2: 5% (Total 4–5: 70%)

ESVS = European Society for Vascular Surgery.

**Table 2:** Preferred formats and enhancements to improve the educational value of the European Society for Vascular Surgery podcasts (*n* = 57).

Format or enhancement	Responses, % (*n*)
Two minute key summary at the end of the episode	68 (39)
Links to further reading in the podcast description	51 (29)
Intended learning objectives at the beginning	37 (21)
Accompanying text information in the episode description	33 (19)
Accompanying visual abstracts	32 (18)
Knowledge quizzes	28 (16)
Episode transcripts	21 (12)
Key takeaway summaries	67 (38)
Reflective prompts (self directed questions)	5 (3)

### Application of Bloom's Taxonomy to podcast content and structure

Bloom's taxonomy was used to evaluate the three selected podcasts' content and structure. One example is provided in the results (Table 3), the other two are attached as a supplement (Supplementary Material S1 and S2). Intended learning outcomes were defined for each podcast, again one in the results (Table 4), and the other two as supplements (Supplementary Material S1 and S2). Across all three episodes, the content primarily focused on lower order cognitive processes, focusing on: remembering, by recalling key guidelines and previous literature; understanding, by explaining the rationale behind clinical recommendations and risk stratification strategies; and applying, by demonstrating how these recommendations may be used in generic or implied clinical contexts (Table 3, Supplementary Material S2 and S3). Higher order cognitive skills, such as analysing and evaluating, were more sporadically addressed, and none of the episodes included explicit activities to support the create domain.

**Table 3:** Bloom's Taxonomy matrix applied to the European Society for Vascular Surgery (ESVS) podcast episode #ESVS2024 – Vascular access pre-op planning and patient selection, presented by Gentile F and Brambilla R; with a length of 14:39 minutes and published on 6 February 2025.

Bloom's cognitive level	Evidence from podcast content	Enhancement suggestions
Remembering	Discusses basic anatomic considerations, definitions of access types (AV fistula *vs*. graft), and patient characteristics; references 2018 ESVS Vascular Access Guidelines	Provide a visual glossary or short downloadable handout summarising key access types and indications
Understanding	Explains rationale of various patient comorbidities and venous characteristics that can lead to failure of vascular access	Include short “pause and reflect” questions, such as: “Why might a patient with diabetes be less suitable for a distal AVF?” or “Would your clinical approach change if a patient had a thick and fibrotic cephalic vein, but with a 2.5–3 mm diameter?”
Applying	Describes how to use guidelines and patient characteristics to choose optimal access strategies	Add a clinical scenario for listeners to apply criteria and decide on access type. Encourage pause before revealing suggested answer
Analysing	Analyses what preventive measures can be implemented for increased vascular access success rates	Include a prompt like: “What does early monitoring mean exactly?” or “In what cases would you perform intra-operative mapping?”
Evaluating	Briefly touches on how MDT decision making may lead to different outcomes depending on local experience.	Add a follow up discussion or reflective question: “In your centre, how does surgical expertise influence your access decisions?”
Creating	Not explicitly supported	Encourage listeners to draft their own pre-operative access planning checklist based on the episode and local guidelines. Could be shared as an example in follow up materials or online community

AVF = arteriovenous fistula; MDT = multidisciplinary team.

**Table 4:** Proposed intended learning outcomes and pedagogical enhancements for the European Society for Vascular Surgery (ESVS) podcast episode #ESVS2024 – Vascular access pre-op planning and patient selection, presented by Gentile F and Brambilla R; with a length of 14:39 minutes and published on 6 February 2025.

Bloom's Cognitive level	**Outcomes and enhancements**
Remembering	Identify the key types of vascular access used in patients requiring haemodialysis (e.g., AVF, grafts, central venous catheters)
	Which is typically considered the first line vascular access for chronic haemodialysis? A) Tunnelled central venous catheter B) AVF (correct answer) C) Synthetic graft D) PICC line
Understanding	Describe the clinical factors influencing patient selection for different vascular access strategies, including comorbidities and vascular anatomy.
	Short answer question: Name three clinical factors that may make a patient less suitable for distal AVF creation.
Apply	Apply current ESVS recommendations to determine an appropriate pre-operative planning strategy for vascular access in a typical clinical scenario.
	Case based question: A 65 year old diabetic man with previous central venous catheters presents for first time vascular access. Duplex ultrasound shows poor radial artery flow bilaterally. According to ESVS guidelines and episode discussion, what would be your next step in planning access?
	Active learning prompt: Sketch or describe a step by step pre-op planning approach based on the podcast discussion.
Analysing	Compare and contrast the advantages and limitations of AVF *vs.* synthetic grafts in different patient populations.
	Fill in the table comparing AVF *vs*. graft:
Evaluating	Evaluate a patient case example and justify the choice of vascular access based on patient specific risk factors and expected outcomes.
	Reflective prompt: Think about a recent case you've encountered. Based on what you've learned, would you have made the same access decision again? Why or why not?

AVF = arteriovenous fistula; PICC = peripherally inserted central catheter.

### Constructive alignment and pedagogical enhancements

**Figure 1:**
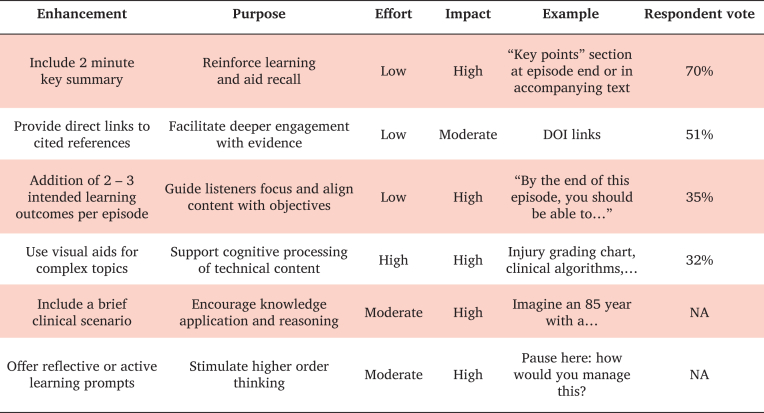
Feasibility map of potential pedagogical enhancements, including anticipated effort, impact and survey respondent vote.

Episode specific ILOs were created and translated into assessment prompts, including short questions, reflective prompts, and structured comparison tasks directly linked to episode content (Table 4, Supplementary Material S2 and S3). Key point summaries condensed episode content into concise, clinically focused take home messages (Table 5, Supplementary Material S2 and S3). Although some of the selected episodes included basic structural support, such as audio transcripts and links to the featured articles, overall, they did not include adjunctive educational materials, such as visual aids, summary tables, or decision making tools. Finally, feasibility mapping showed that several enhancements, particularly the creation of key point summaries, providing direct links to cited references and inclusion of two to three ILOs per episode, were categorised as low implementation effort with moderate and high anticipated educational impact and aligned with listener support identified in the survey (Fig. 1).

**Table 5:** Proposed key point summary for the European Society for Vascular Surgery (ESVS) podcast episode #ESVS2024 – Vascular access pre-op planning and patient selection, presented by Gentile F and Brambilla R; with a length of 14:39 minutes and published on 6 February 2025.

Arteriovenous fistula first approach	Arteriovenous fistula remains the first line recommendation for haemodialysis access due to its superior patency and lower infection risk Patient selection is crucial, particularly regarding vessel quality and comorbidities
Role of pre-operative duplex ultrasound	Duplex scanning is essential for assessing vessel suitability and planning fistula creation Common anatomic pitfalls and threshold values for vessel diameter were briefly discussed
Patient specific considerations	Comorbidities such as diabetes, peripheral vascular disease, and previous catheter placements influence access strategy
Team based decision making and integrating patient preference	Emphasis on multidisciplinary collaboration between nephrologists, vascular surgeons, and radiologists to optimise planning and outcomes Consider patient preference and lifestyle when choosing access site and type

## DISCUSSION

This evaluation demonstrates that the ESVS educational podcasts are a well received and widely used learning resource among vascular professionals. Survey respondents reported high levels of satisfaction, strong perceptions of clarity and evidence based content, and high engagement with podcast hosts and guests. Importantly, more than 70% indicated improved understanding of vascular topics, and over three quarters reported applying podcast derived knowledge in their daily practice, underscoring the relevance and practical utility of the series.

Bloom's Taxonomy revealed that episodes are very good at targeting lower order cognitive skills, which are appropriate for a short, focused, audio based educational format. However, there are opportunities to integrate additional elements that could strengthen higher order cognitive engagement, particularly for episodes with a more explicit educational intent, such as guideline interpretation, clinical scenarios, or discussions of complex risk benefit reasoning.^19^ Survey feedback was closely aligned with the pedagogical analysis, with listeners supporting several low effort, high impact enhancements (Fig. 1), such as concise key summaries, linked reading materials, and intended learning outcomes, which could be readily integrated into the current production workflow. While more resource intensive features, such as visual abstracts or structured clinical scenarios, were less frequently selected, they may be beneficial for specific types of content, including guideline episodes, injury classifications, or complex decision making topics.

These results are consistent with a recent scoping review of 62 studies examining podcast use in medical education, which showed that learners valued key summaries, credible source material, and quiz based reinforcement.^5^^,^^20^^,^^21^ However, as highlighted by Kelly *et al.*, most of the included studies assessed only initial learner response (Kirkpatrick Level 1), with relatively few evaluating knowledge retention (Level 2), behavioural change (Level 3), or system level outcomes (Level 4). The lack of data on patient outcomes or practice change underscores the need for future research into the long term educational and clinical impact of podcasts. The study by Dmytryshyn *et al.* on knowledge retention in vulvovaginal disease podcasts, for example, found that while factual knowledge was retained after two weeks, participants’ confidence in applying that knowledge declined with time, highlighting the importance of integrating new learning into realistic clinical contexts.^22^

The findings from this study highlight an opportunity to more closely align the ESVS podcasts with other educational initiatives. Selected episodes could be used as preparatory learning tools for ESVS Academy workshops, academic pathways, webinars, and even dedicated fellowship programs. If paired with clear learning objectives and brief assessments, such episodes could even be considered for future continued medical education accreditation, as implemented in some medical specialties. This would not apply to all episodes but could meaningfully enhance those with a stronger educational focus.

Finally, the structured application of pedagogical frameworks could be extended beyond the ESVS vascular surgery podcasts to other ESVS educational initiatives. The ESVS Academy, which has transformed the Annual Meeting by replacing some of the traditional lectures with hands on workshops and interactive case discussions, already reflects the Society's commitment to outcome driven learning and minimisation of commercial bias.^23^^,^^24^ Embedding clear learning objectives and aligning activities with progressive cognitive goals could further strengthen the impact of Academy sessions, certification programs, and academic pathways. Integrating such frameworks across the educational portfolio may enhance learner engagement and consistency across the rapidly expanding range of ESVS educational activities.

This study had several limitations. It focused on a sample of three episodes, selected to represent diversity in format and content. While this allowed for meaningful analysis, the findings may not be generalisable across the entire podcast series. The framework based qualitative approach was not supplemented with formal post-assessment testing, limiting conclusions about objective learning outcomes. Furthermore, considering the high number of podcast downloads, the response rate (*n* = 57) may be considered quite modest, so these results should be interpreted with caution. Additionally, 5% of respondents reported not having listened to the ESVS podcasts, increasing the uncertainty of some of the presented estimates. Future studies could incorporate both pre-podcast and post-podcast listener analytics, quantitative assessments, or structured pilot testing of enhancements such as reflective questions and visual aids.

### Conclusion

The ESVS vascular surgery podcasts represent a valued and accessible educational resource, with a good educational rating. This evaluation highlights opportunities, supported by both theory and listener feedback, to strengthen their sustained pedagogical impact, potentially improving knowledge retention and clinical applicability.

## Funding

No funding was provided.

## Conflicts of interest

None.
