# Supplementary material for: Applying Constructive Alignment to Enhance the Educational Structure of the European Society for Vascular Surgery Podcasts
Source: EJVES Vasc Forum. 2026 Feb 13;65:100–6. doi: 10.1016/j.ejvsvf.2026.02.002 (PMC13049894; doi:10.1016/j.ejvsvf.2026.02.002)
Supplement: Multimedia component 1 [file mmc1.docx]

**Supplementary Material S1**

**Editor's Choice: Radiation Protection and Pregnancy
Presenters**: F. Jennifer M J Jongen
**Length**: ca. 13 min; 24^th^ April 2025
**Type**: Clinically applicable scenario

| **Bloom’s Cognitive Level** | **Evidence from Podcast content** | **Enhancement suggestions** |
| --- | --- | --- |
| **Remembering** | Cites the 2023 ESVS Radiation Safety Guidelines and a recent Annals of Surgery publication highlighting pregnancy outcomes in surgical staff. | Include full reference citations in the episode description (e.g., Modarai B et al., Eur J Vasc Endovasc Surg 2023;65:171–222; Tatarian T et al., Ann Surg 2024). Ensure that the discussed article is open access or provide access via institutional login or DOI. |
| **Understanding** | Explains the rationale for the Dutch study: absence of uniform national policy on radiation protection for pregnant clinicians. Notes that 20% of women altered reproductive plans due to radiation exposure risks. | Embed a brief "pause-and-reflect" moment within the episode: e.g., “How would you feel if you or your partner had to choose between career progression and pregnancy due to radiation exposure risks?” |
| **Applying** | Discusses how lack of national guidance negatively affects clinicians’ sense of safety, confidence, and autonomy. | Add a clinical scenario prompt for reflection: “Imagine you’re advising a trainee who is early in pregnancy and anxious about their IR rotation – how would you navigate this conversation?” |
| **Analyzing** | Explores how pregnancy can disrupt training progression in vascular surgery and IR, and reveals differing perceptions between the two fields. | Ask learners to list three adaptive changes to support pregnant trainees, such as: alternative rotations, scheduling flexibility, and simulation-based training. |
| **Evaluating** | Emphasizes the need for individualized approaches: better communication, improved PPE, and inclusion of this issue in workplace conversations. | Invite listeners to critically appraise their institution's current approach. What works? What are the gaps? Who should lead the conversation? |
| **Creating** | Not directly addressed in podcast. | Encourage learners to design an alternative training schedule for pregnant colleagues that avoids radiation exposure but ensures continued skill development. This could include simulation labs, academic projects, and tele-supervised clinical decision-making. |

***Intended Learning Outcomes (ILOs)***

By the end of this episode, the learner should be able to:

1. **Identify** recent and relevant publications addressing radiation protection and pregnancy during surgical and interventional radiology training.
   (Bloom level: Remembering)
2. **Explain** the rationale for investigating radiation exposure and its influence on pregnancy planning and decision-making among clinicians.
   (Bloom level: Understanding)
3. **Reflect** on how the absence of national or institutional policy affects the professional choices and security of pregnant healthcare providers.
   (Bloom level: Applying)
4. **Compare** the differing perspectives and workplace experiences of vascular surgeons and interventional radiologists regarding pregnancy-related radiation risk.
   (Bloom level: Analyzing)
5. **Evaluate** potential strategies and institutional pathways that may improve communication, support structures, and decision-making during pregnancy.
   (Bloom level: Evaluating)
6. **Propose** a personalized, low-radiation training plan for a 9–18 month period that maintains core educational and skill development goals while minimizing exposure.
   (Bloom level: Creating)

**Key findings**: The references discussed during the podcast were not explicitly cited or listed in the episode description. Additionally, the featured article is not open access and can only be accessed through institutional or personal subscriptions. Including a direct link to the survey and inviting listeners to participate may have enhanced engagement. Furthermore, listing relevant organizations or professional networks related to pregnancy and radiation safety would have provided valuable opportunities for further reading and support.
