# Supplementary material for: Applying Constructive Alignment to Enhance the Educational Structure of the European Society for Vascular Surgery Podcasts
Source: EJVES Vasc Forum. 2026 Feb 13;65:100–6. doi: 10.1016/j.ejvsvf.2026.02.002 (PMC13049894; doi:10.1016/j.ejvsvf.2026.02.002)
Supplement: Multimedia component 2 [file mmc2.docx]

**Supplementary Material S2**

**#ESVS2024 – Q&A ESVS Vascular Trauma Guidelines: Pt 2
Presenters: P. Vikatmaa & J.B. Ricco
Length**: 31.16 minutes; March 20^th^, 2025
**Type**: Evidence-based discussion on a recent Editor’s Choice publication

| **Bloom’s Cognitive Level** | **Evidence from Podcast content** | **Enhancement suggestions** |
| --- | --- | --- |
| **Remembering** | Recalls ESVS injury grading system for cervical artery trauma (Grades I–X) and BTAI classification for blunt thoracic aortic injury (minimal, intimal tear, pseudoaneurysm, rupture). | Provide summary tables and schematics in episode description or as downloadable handouts to reinforce classification systems. |
| **Understanding** | Explains the rationale for non-operative management in stable carotid/vertebral injuries and the timing of repair in blunt aortic trauma. | Add in-episode prompts such as: “Why might antithrombotic therapy be preferred over immediate intervention in some BCVI cases?” |
| **Applying** | Applies guidelines to clinical cases: e.g., asymptomatic Grade II carotid injury → antiplatelet therapy and selective endovascular treatment; minimal aortic injury → observation with serial imaging. | Include narrated case examples: “A 40-year-old with head trauma and CTA showing vertebral dissection—what next?” |
| **Analyzing** | Differentiates between injuries requiring medical therapy, endovascular repair, or surgical intervention, based on grade and associated injuries. Analyses how full heparinization should be performed in these patients. | Suggest flowchart-building exercises: “Map management pathways for Grade I vs. Grade III cervical artery injury in blunt trauma.” |
| **Evaluating** | Critically appraises the timing and method of repair for blunt thoracic aortic injury based on patient stability, trauma burden, and imaging. Implications of the role of hemodynamic stability of endograft oversizing. | Add prompts for institutional comparison: “How does your center manage BTAI? What are your triggers for endovascular repair?” |
| **Creating** | Encourages development of integrated trauma protocols incorporating grade-based decision-making and imaging timelines. | Invite listeners to draft or adapt their local blunt vascular trauma algorithm, including imaging, triage, and intervention steps. |

***Intended Learning Outcomes (ILOs)***

After listening to this episode, the listener should be able to:

1. **Identify the ESVS grading systems for carotid/vertebral artery injuries and classification of blunt thoracic aortic injuries. (Remembering)**
2. **Explain the rationale for conservative management in low-grade cervical artery injuries and delayed intervention in stable blunt thoracic aortic injury. (Understanding)**
3. **Apply guideline-based recommendations to select appropriate medical, surgical, or endovascular therapy for cervical and thoracic vascular injuries. (Applying)**
4. **Analyze how patient status, imaging findings, and trauma burden guide treatment pathways in neck and thoracic vascular trauma. (Analyzing)**
5. **Evaluate the evidence and decision-making process for timing of TEVAR in BTAI and use of antithrombotic therapy in BCVI. (Evaluating)**
6. **Create a structured trauma care algorithm for managing blunt cervical and thoracic vascular injuries at the institutional level. (Creating)**

**Specific suggestions for enhancement**: Provide visual adjuncts, including a list of recommendations discussed – so that listeners can ‘prepare’ before listening to this episode by reading one by one the specific recommendations discussed in this section. Provide links or visual adjuncts of different scales discussed for improved following and comprehension (Denver screening criteria for blunt cervical vascular injury).

**# Key point summary:**

- **Shift from Anatomical Zones to Injury Grades**: The 2024 guidelines replace the outdated zone I–III classification with a **morphologic injury grading system** (Grades I–V), which better aligns with imaging findings and treatment strategies.
- **Grade-Based Classification Focuses on Lesion Characteristics**:
- **Grade I**: Partial wall injury; Normal external wall contour
- **Grade II**: Complete wall injury, Abnormal external wall contour, Contained bleeding
- **Grade III**: Complete wall injury; Uncontained haemorrhage
- **Grade IV**: Occlusion
- **Blunt Thoracic Aortic Injury (BTAI)**
- Injuries are categorized from **minimal intimal injury to full-thickness rupture**.
- **TEVAR** is the preferred modality for repair, replacing open thoracotomy in nearly all cases.
- **Stable patients with minor injuries** (minimal or isolated intimal flaps) may be managed conservatively with **BP control and surveillance**.
- **Timing of TEVAR** is often **delayed** (24–72 hrs) in polytrauma patients to prioritize stabilization of other life-threatening injuries.
- Emphasis is placed on **early imaging**, BP management, and multidisciplinary coordination with trauma, anesthesia, and ICU teams.
