# Supplementary material for: Applying Constructive Alignment to Enhance the Educational Structure of the European Society for Vascular Surgery Podcasts
Source: EJVES Vasc Forum. 2026 Feb 13;65:100–6. doi: 10.1016/j.ejvsvf.2026.02.002 (PMC13049894; doi:10.1016/j.ejvsvf.2026.02.002)
Supplement: Multimedia component 3 [file mmc3.docx]

**Supplementary Table S1. Demographics of survey respondents (*n* = 57).**

| **Characteristic** | **Category** | **% (n)** |
| --- | --- | --- |
| **Gender** | Male | 61% (35) |
|  | Female | 39% (22) |
| **Profession** | Vascular surgeon | 90% (51) |
|  | Trainee / resident / early-career clinician | 11% (6) |
| **Years of experience** | 0–5 years | 37% (21) |
|  | 6–10 years | Not reported |
|  | 11–20 years | Not reported |
|  | >20 years | Not reported |
| **Continent of practice** | Europe | 74% (42) |
|  | Africa | 18% (10) |
|  | Other (Americas / Asia / Oceania) | 9% (5) |
| **Listening habits** | Frequent listeners | 54% (31) |
|  | Occasional listeners | 30% (17) |
|  | Rare / infrequent | 16% (9) |
